# tDCS and neurofeedback in ADHD treatment

**DOI:** 10.3389/fnsys.2025.1444283

**Published:** 2025-09-18

**Authors:** Alexandra Bernadotte, Oksana Zinchenko

**Affiliations:** ^1^Department of Information Technologies and Computer Sciences, The National University of Science and Technology MISIS, Moscow, Russia; ^2^Laboratory of Analysis of Semantics, Centre for Language and Semantics Technologies, HSE University, Moscow, Russia; ^3^Institute of Artificial Intelligence, M. V. Lomonosov Moscow State University, Moscow, Russia; ^4^Centre for Cognition and Decision Making, Institute for Cognitive Neuroscience, HSE University, Moscow, Russia

**Keywords:** ADHD, tDCS, neurofeedback, EEG, transcranial alternating current stimulation (tACS)

## Abstract

Attention deficit hyperactivity disorder (ADHD) stands as one of the most prevalent neurodevelopmental disorders, affecting millions worldwide. While traditional pharmacological interventions have been the cornerstone of ADHD treatment, emerging novel methods such as transcranial Direct Current Stimulation (tDCS) and neurofeedback offer promising avenues for addressing the multifaceted challenges of ADHD management. This review paper critically synthesizes the current literature on tDCS and neurofeedback techniques in ADHD treatment, elucidating their mechanisms of action, efficacy, and potential as adjunct or alternative therapeutic modalities. By exploring these innovative approaches, this review aims to deepen our understanding of neurobiological underpinnings of ADHD and pave the way for more personalized and effective interventions, ultimately enhancing the quality of life for individuals grappling with ADHD symptoms.

## 1 Introduction

### 1.1 ADHD prevalence

Recent estimates (2020–2023) suggest that Attention Deficit Hyperactivity Disorder, or ADHD, globally affects 5%–7.2% of youth and 2.5%–6.7% of adults (Posner et al., 2020; Song et al., 2021; Simon et al., 2009). Recent estimates indicate that prevalence is even higher in children and could reach 8.7% (Bozinovic et al., 2021). A more recent epidemiological update by Ayano et al. (2023) analyzed data across multiple cohorts and geographic regions, reporting a global childhood ADHD prevalence estimate of 8 %, with regional variations linked to socioeconomic factors and healthcare accessibility. This suggests an increasing recognition of ADHD diagnoses and highlights the need for scalable, non-pharmacological interventions. The upward trend may also reflect improved screening tools and greater awareness, though diagnostic inflation cannot be ruled out.

Although it has long been conceptualized as a disorder of childhood, up to 90% of children with ADHD continue to experience symptoms into adulthood (Sibley et al., 2022). These symptoms can significantly impact various aspects of their lives, including academic performance, employment, and relationships. It is important for individuals with ADHD to receive appropriate diagnosis and treatment throughout their lifespan to effectively manage their symptoms and improve their overall quality of life.

## 2 ADHD diagnostics

There are ADHD with predominately inattentive domain, with predominately hyperactive-impulsive domain, or with combined domain. At least five symptoms in either domain are required to meet the adult diagnostic criteria and at least the presence of six or more symptoms in either domain are required for children (Posner et al., 2020). In the International Classification of Diseases-11 requires a persistent pattern (e.g., at least 6 months) of inattention symptoms and/or a combination of hyperactivity and impulsivity symptoms^1^ (see Table 1).

**TABLE 1:** Attention deficit hyperactivity disorder (ADHD) criteria in the International Classification of Diseases-11.

Inattention	• Difficulty sustaining attention to tasks that do not provide a high level of stimulation or reward or require sustained mental effort; lacking attention to detail; making careless mistakes in school or work assignments; not completing tasks. • Easily distracted by extraneous stimuli or thoughts not related to the task at hand; often does not seem to listen when spoken to directly; frequently appears to be daydreaming or to have mind elsewhere. • Loses things; is forgetful in daily activities; has difficulty remembering to complete upcoming daily tasks or activities; difficulty planning, managing and organizing schoolwork, tasks and other activities.
Hyperactivity/ impulsivity	• Excessive motor activity; leaves seat when expected to sit still; often runs about; has difficulty sitting still without fidgeting (younger children); feelings of physical restlessness, a sense of discomfort with being quiet or sitting still (adolescents and adults). • Difficulty engaging in activities quietly; talks too much. • Blurts out answers in school, comments at work; difficulty waiting turn in conversation, games, or activities; interrupts or intrudes on others conversations or games. • A tendency to act in response to immediate stimuli without deliberation or consideration of risks and consequences (e.g., engaging in behaviors with potential for physical injury; impulsive decisions; reckless driving)

## 3 Current treatment approaches for ADHD. Pharmacological and non-pharmacological approaches

Current therapeutic approaches for ADHD are associated with significant clinical challenges. Although studies demonstrated the effectiveness of stimulant drugs (e.g., methylphenidate) to reduce ADHD symptoms, such as hyperactivity, poor focus and impulsivity, there are many aspects of concern, such as: tachyphylaxis, particularly when chronically used (Volkow and Swanson, 2013); significant side effects (Feldman and Reiff, 2014; Volkow and Swanson, 2013); the risks of abuse and addiction (Shier et al., 2013); and unclear long-term cost-effectiveness (Gilmore and Milne, 2001). Furthermore, there is a growing concern about the overdiagnosis and overprescription of stimulant drugs for ADHD, as well as the potential for misdiagnosis and the need for alternative treatment options.

Additionally, research has shown that psychosocial non-pharmacological approaches, such as behavioral therapy and parent training, can also be effective in managing ADHD symptoms and improving overall functioning (Taylor et al., 2004).

Hardware non-pharmacological treatments currently available are: transcranial magnetic stimulation (TMS), transcranial direct current stimulation (tDCS), neurofeedback training. Moreover, non-pharmacological treatments using artificial intelligence are currently gaining momentum.

However, non-pharmacological approaches are recommended as part of the multimodal approach. Multimodal combined approach was superior to pharmacological alone in improving functional outcomes (The MTA Cooperative Group, 1999).

### 3.1 Pharmacological approaches

Pharmacological treatment remains the first-line intervention for moderate to severe ADHD, with stimulants—primarily methylphenidate (MPH) and amphetamine (AMPH) derivatives—being the most commonly prescribed. These agents work primarily by inhibiting dopamine and norepinephrine reuptake, thereby enhancing neurotransmission in fronto-striatal circuits implicated in attention and executive function (Arnsten, 2009).

The main approach of treating ADHD with medication involves the administration of stimulant drugs, such as methylphenidate (e.g., Ritalin, Concerta, Equasym, Medikinet), D-amphetamines (e.g., Adderall), as well as non-psychostimulants, such as atomoxetine and guanfacine. These drugs function by augmenting the amounts of specific neurotransmitters in the brain, thereby enhancing concentration and diminishing impulsiveness. They have demonstrated efficacy in controlling symptoms of ADHD in numerous individuals. However, while amphetamines also appear to be effective in alleviating the primary symptoms of ADHD in the short term, they are also linked to several negative occurrences (such as reduced appetite, sleeplessness, stomach pain, nausea, vomiting, anxiety) (see Punja et al., 2012 for a review).

Methylphenidate is usually prescribed due to its observed positive impact in mitigating the primary symptoms of heightened hyperactivity, impulsivity, and inattention in children and adolescents diagnosed with ADHD (Storebø et al., 2015). Methylphenidate has shown robust short-term efficacy across numerous randomized controlled trials, with effect sizes consistently ranging from 0.6 to 0.9 for core ADHD symptoms (Faraone and Buitelaar, 2010; Storebø et al., 2015). Common side effects include reduced appetite, insomnia, irritability, and, less frequently, cardiovascular concerns (Wolraich et al., 2011). Long-acting formulations such as Concerta and Equasym XL have been developed to reduce dosing frequency and improve compliance (Coghill et al., 2013). Methylphenidate has affinity for both dopamine and noradrenaline transporters, effectively inhibiting their function and resulting in elevated levels of noradrenaline and dopamine in the synaptic cleft (Heal and Pierce, 2006; Volkow et al., 2012). It is believed that this leads to an overall increase in the firing rate by enhancing the transmission of dopamine and noradrenaline neurotransmitters, which, when the prefrontal cortex is targeted, can lead to the improvement in executive functions (Arnsten and Dudley, 2005). Amphetamine derivatives (e.g., Adderall, Vyvanse) also show high efficacy, with similar or slightly greater effect sizes than MPH in some comparative studies (Faraone et al., 2004; Clemow and Bushe, 2015). However, they may carry a higher risk of emotional dysregulation and are more often associated with abuse potential, particularly in adolescent populations (Lakhan and Kirchgessner, 2012).

When mentioning methylphenidate and amphetamines, both of which provide almost instant relief from symptoms, it is important to note that some studies have also investigated other treatment approaches, such as non-stimulants like atomoxetine and α2-adrenoceptor agonists (guanfacine) (see Sallee et al., 2013 for a review). Non-stimulants such as atomoxetine, guanfacine, and clonidine offer alternatives for patients with contraindications or poor tolerance to stimulants. Atomoxetine, a selective norepinephrine reuptake inhibitor, has shown moderate efficacy (effect sizes around 0.6) and a more gradual onset of action (Michelson et al., 2001). Guanfacine and clonidine, α2A-adrenergic agonists, are particularly useful in cases with comorbid sleep or tic disorders (Banaschewski et al., 2006). Atomoxetine and guanfacine are non-controlled drugs that have been authorized for use in several European nations and the United States. They are specifically approved for treating ADHD in children aged 6 years and older. However, its effect in lowering ADHD symptoms typically takes several weeks to become apparent (Drechsler et al., 2020).

Long-term outcomes remain a subject of debate. While pharmacotherapy effectively reduces symptom severity, its benefits on academic performance, social functioning, or emotional development are less clear (Molina et al., 2009). Moreover, adherence to medication is suboptimal, with discontinuation rates exceeding 50% after the first year in some cohorts (Gajria et al., 2014). Given the heterogeneity of ADHD and the potential adverse effects of pharmacological treatments, multimodal approaches combining medication with behavioral or cognitive interventions are increasingly advocated (The MTA Cooperative Group, 1999).

Despite short-term efficacy, the long-term safety and developmental consequences of stimulant use remain under scrutiny. Volkow et al. (2012) demonstrated that methylphenidate increases dopamine availability in the ventral striatum, which correlates with symptom relief. However, this dopaminergic modulation raises concerns about long-term neuroadaptive changes, particularly in adolescents with prolonged exposure. Potential risks include growth suppression, altered reward processing, and emotional dysregulation. These risks reinforce the need for individualized medication planning, regular monitoring, and evaluation of non-pharmacological alternatives.

### 3.2 Hardware non-pharmacological: transcranial direct current stimulation

Transcranial direct current stimulation (tDCS) has emerged as an effective tool for modulating spontaneous neural network excitability (Brunoni et al., 2012; DaSilva et al., 2011). tDCS yields low-intensity electrical current to modulate targeted brain regions, such that it can increase or decrease excitability of the neural tissue. This low-intensity direct current is thought to modulate neuronal activity, leading to changes in brain function and behavior. The use of tDCS has been explored in various fields, including neuroscience, psychology, and medicine. Researchers have investigated its potential applications in treating psychiatric disorders such as depression (Jog et al., 2019), anxiety (Stein et al., 2020), and addiction (Chen et al., 2020). It has also been studied as a means to enhance cognitive abilities like memory (Galli et al., 2019) and attention (Reteig et al., 2017).

One of the advantages of tDCS is its safety profile compared to other brain stimulation techniques. Compared to TMS, tDCS is posed as a relatively safe method with no serious adverse effects reported in 747 sessions (Salehinejad et al., 2020b). The low intensity of the electrical current used minimizes the risk of adverse effects or tissue damage. Furthermore, tDCS is relatively inexpensive and portable, making it an attractive option for both research and clinical settings. Despite these advantages, there are still several challenges associated with tDCS research (Ho et al., 2022). One limitation is the lack of standardized protocols for electrode placement and stimulation parameters. This variability makes it difficult to compare results across studies or establish clear guidelines for clinical practice.

Transcranial direct current stimulation (tDCS) is a established method for altering cortical excitability with clinical implications. It has been increasingly used in neurodevelopmental disorders, especially attention-deficit hyperactivity disorder (ADHD), and its efficacy (based on effect size calculations), safety, and stimulation parameters have been systematically reviewed in 2020 (Salehinejad et al., 2020b).

The main target is either cognitive deficits (response inhibition, working memory, attention, and cognitive flexibility) or clinical symptoms (e.g., impulsivity and inattention). According to systematic review (Salehinejad et al., 2020b), partial improvement has been seen in 10 out of 14 eligible for review studies. Studies that applied tDCS in ADHD patients have shown beneficial effects on interference control (Breitling et al., 2016), functional connectivity (Cosmo et al., 2015, Sotnikova et al., 2017), different aspects of executive functions (Soltaninejad et al., 2019; Bandeira et al., 2016; Nejati et al., 2020) and general ADHD symptoms (Cachoeira et al., 2017; Soff et al., 2017). In addition to that, more recent systematic review by Cosmo et al. (2020) significant significant improvements in attention, inhibitory control were paralled with increased brain connectivity following use of active tDCS. However, no robust effects of tDCS have been observed in memory domain (Nejati et al., 2020; Soff et al., 2017; Sotnikova et al., 2017).

A mathematical model was utilized to investigate the impact of transcranial direct current stimulation (tDCS) on cortical connections in individuals with attention deficit hyperactivity disorder (ADHD), with a specific focus on neurophysiological causes. According to the study conducted by Cosmo et al. (2015), functional connectivity measured by EEG prior to and immediately following a 20 min transcranial direct current stimulation (tDCS) session over the left dorsolateral prefrontal cortex (DLPFC) significantly altered. The findings suggested an observed augmentation in cortical connectivity subsequent to anodal stimulation within the stimulated region and its associated factors, indicating a propagation of the modulatory effects (Cosmo et al., 2015).

These findings suggest that active tDCS may be a successful intervention for improving attention and inhibitory control in individuals with ADHD. However, further research is needed to better understand the effects of tDCS on memory function in this population.

#### 3.2.1 tDCS protocols: intensity and target areas

The intensity of tDCS protocols varies in studies on ADHD children and adults. The intensity most frequently associated with the significant behavioral improvement is 2 mA in adults, while the studies on children mostly employ 1 mA. It is important to note that the optimal intensity for tDCS protocols may differ based on individual factors such as age and severity of ADHD symptoms. Meta-analytic data suggest that stimulation intensity interacts with age and symptom target in influencing efficacy. Salehinejad et al. (2020a,b) found that studies using 1 mA stimulation in children showed comparable or superior outcomes on attention tasks to 2 mA studies in adults, possibly due to age-related differences in skull thickness and cortical excitability. Moreover, the effects appear more robust when combined with optimized electrode placement and task-relevant engagement during stimulation. Additionally, future research should investigate the long-term effects of different intensity levels to determine the most effective approach for improving memory function in individuals with ADHD.

Neuroimaging studies have revealed the presence of structural and functional modifications in various regions of the brain, such as the dorsolateral prefrontal cortex and inferior frontal gyrus, among persons diagnosed with ADHD (Nickel et al., 2018; Depue et al., 2010; Arnsten, 2009). The left and right DLPFC are the regions most often targeted, and anodal tDCS the protocol most often applied. Recent studies either specifically reported the selection of left (Soltaninejad et al., 2019) or right DLPFC (Salehinejad et al., 2022) as a target area, or specified the anatomic location in right inferior frontal gyrus (Breitling et al., 2016). Few studies targeted posterior regions, such as the right posterior parietal cortex (Salehinejad et al., 2020b). The application of anodal transcranial direct current stimulation (tDCS) targeting the left dorsolateral prefrontal cortex (dlPFC) has been found to alleviate symptoms associated with ADHD. Additionally, this form of stimulation has been shown to enhance memory consolidation, inhibitory control, selective attention, working memory and interference control, reward processing, and sustained attention (Nejati et al., 2022). Majority of the studies applied tDCS in awake state; however, several studies reported using oscillatory tDCS during slow wave sleep. Prehn-Kristensen et al. (2014) demonstrated an improvement of declarative memory performance on the next day as well as improved reaction times in a go/nogo task in children with ADHD (Munz et al., 2015).

Few studies report the usage of HD-tDCS: high-definition tDCS, which can potentially allow to stimulate the target area more precisely by achieving focality of the stimulation. For HD-tDCS small disk electrodes are placed in a 4 × 1 configuration with the stimulation electrode being surrounded by four reference electrodes in a ring-like pattern (Datta et al., 2009). The HD-tDCS 4 × 1 ring configuration allows for more focal stimulation by concentrating the current density at the central electrode while minimizing peripheral diffusion. Electric field modeling by Datta et al. (2009) demonstrated that this configuration enhances spatial precision compared to conventional sponge montages, making it particularly suitable for targeting small cortical areas such as the right inferior frontal gyrus implicated in inhibitory control. However, methodological studies which employed both conventional tDCS and HD-tDCS in ADHD, showed no critical improvement in cognitive domains after using HD-tDCS in comparison to conventional montage (Breitling et al., 2020). A major limitation across studies remains the small sample sizes, often ranging from 15 to 30 participants, which limits statistical power and generalizability. Breitling et al. (2020) utilized only 20 participants in their HD-tDCS study, which hinders the interpretation of subgroup effects such as age, symptom profile, or comorbidities. Future trials must ensure adequately powered, age-stratified samples and include replication cohorts to establish the robustness of findings.

These findings suggest that while HD-tDCS may offer the advantage of precise targeting, it may not necessarily lead to significant cognitive improvements in individuals with ADHD. Further research is needed to explore the potential benefits of HD-tDCS in this population and identify factors that may influence its effectiveness.

#### 3.2.2 Other transcranial electrical stimulation modalities: tACS and tRNS

In addition to tDCS, other forms of transcranial electrical stimulation (tES) have been investigated in ADHD, particularly transcranial alternating current stimulation (tACS) and transcranial random noise stimulation (tRNS). tACS modulates neuronal oscillations by delivering sine-wave currents at specific frequencies, thus entraining intrinsic brain rhythms. This is particularly relevant in ADHD, where dysregulation of theta and beta band activity is prominent (Arns et al., 2013).

Recent studies suggest that theta-frequency tACS over the frontal cortex can enhance cognitive control in healthy adults (Vosskuhl et al., 2018), and pilot studies are underway in pediatric ADHD populations. tRNS, on the other hand, delivers a random, high-frequency current that can increase cortical excitability through stochastic resonance. Looi et al. (2017) demonstrated that multi-session tRNS improved arithmetic performance in children with ADHD. Brevet-Aeby et al. (2016) reported improvements in working memory after tRNS in adolescents with attention problems.

While research is still nascent, these modalities offer promising alternatives or adjuncts to tDCS and warrant inclusion in future comparative studies on neuromodulation strategies for ADHD.

### 3.3 Hardware non-pharmacological: neurofeedback

#### 3.3.1 Neurofeedback architecture

**FIGURE 1:**
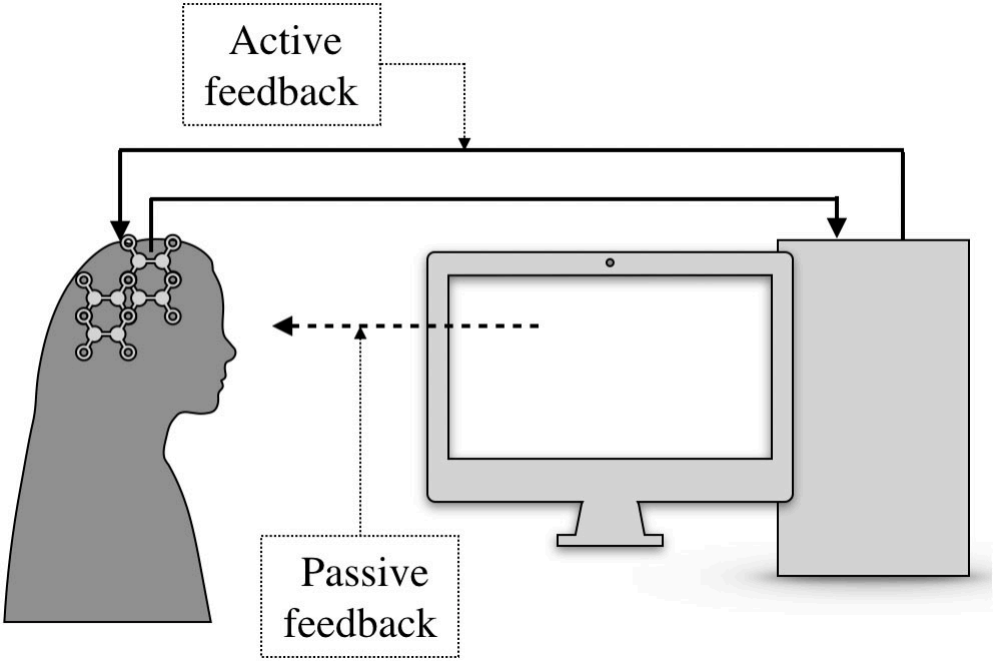
Architecture of neurofeedback systems showing signal acquisition (System 1), signal processing (System 2).

**FIGURE 2:**
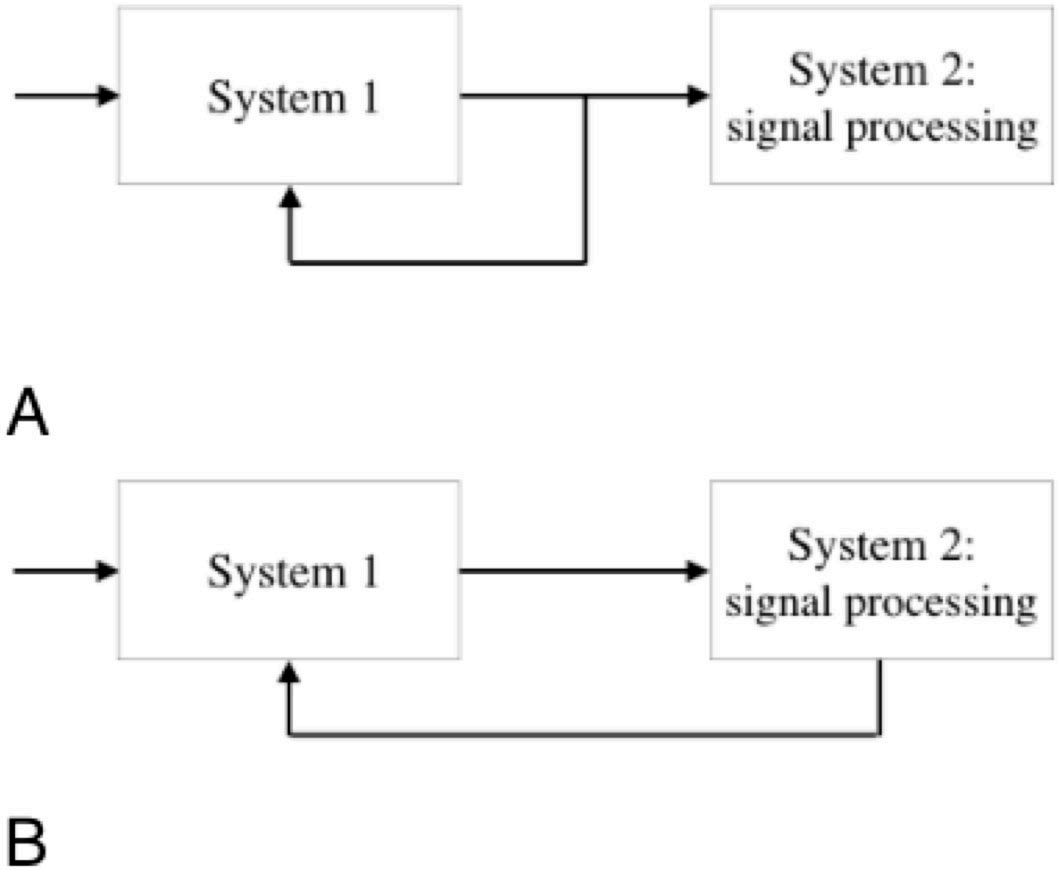
Examples of feedback modalities. Passive feedback: user receives sensory output (e.g., visual or auditory). Active feedback: user engages in real-time modulation of brain activity via training. **(A)** System 1 connected to System 2 with a feedback from System 1. **(B)** System 1 connected to System 2 with a feedback from System 1 to System 2.

Feedback is a system, which outputs are passed back as inputs within a loop (see Figure 1). The implementation of the feedback can be carried out with or without processing of the output information. Neurofeedback is usually called brain-computer interfaces (BCI). For neurofeedback in BCI, the only second type of feedback with processing can be used. The computer always converts the signal from the BCI into a form that the brain can understand. The choice of waveform (modality) depends on whether active or passive feedback can be used (Figure 2).

Passive neurofeedback is implemented through the senses, when a person sees or hears the result of her/his signals emitted by the brain through the BCI. While active feedback is processed through direct interaction with the brain. This can be achieved using implanted electrodes or by changing the electromagnetic field over the head. Whatever the feedback, it always requires the user to actively participate in the learning process and focus (Kalokairinou et al., 2022; Leem et al., 2021; Vorontsova et al., 2021; Bernadotte, 2022; Zubov et al., 2021; Vorontsova et al., 2023).

The most popular type of brain signal modality is the recording of electromagnetic signals using electroencephalography (EEG). It is this type of signal modality that makes it possible to build a neurofeedback that is safe, affordable and easy to use (Kalokairinou et al., 2022; Leem et al., 2021, Vorontsova et al., 2021; Bernadotte, 2022). In clinical psychology, EEG-based BCI can be used to treat neuroses, panic disorders and attention disorders (ADHD and ADD) with promising results (Chung et al., 2022; Neurofeedback Collaborative Group, 2023; Neurofeedback Collaborative Group, 2021).

#### 3.3.2 Neurofeedback and ADHD: EEG-signs of ADHD

The rationale for using EEG-based BCI to treat these conditions using feedback is based on observations of EEG differences between individuals with ADHD and controls. However, the EEG signs of ADHD change with age (Markovska-Simoska and Pop-Jordanova, 2017; Adamou et al., 2020). This may be explained by brain reorganization, including synaptic pruning, during growth and aging.

Increased theta waves (4–8 Hz) in patients with ADHD are associated with focused attention (Angelidis et al., 2016). The theta EEG-signs of ADHD are most pronounced in children (Markovska-Simoska and Pop-Jordanova, 2017; Adamou et al., 2020). Alpha waves (8–10 Hz) showed increased absolute power and decreased relative power in ADHD in adulthood (Koehler et al., 2009; Bresnahan and Barry, 2002; Adamou et al., 2020). Beta waves (12–25 Hz) are not unique to adult ADHD and may not be useful in differentiating adult ADHD from the control group (Koehler et al., 2009; Bresnahan and Barry, 2002; Adamou et al., 2020). This is actually unexpected, since beta waves are associated with brain activity and anxiety (Al-Ezzi et al., 2020). The gamma wave change turned out to be the most stable when it comes to analyzing scientific papers. Reductions in gamma bands (30–50 Hz) were inversely correlated with ADHD severity (Adamou et al., 2020; Barry et al., 2010; Dupuy et al., 2014).

In addition to using the analysis of a particular frequency range, the frequency ratio is also used. Theta/beta ratio increases in ADHD and ADHD-specific pharma remedies stabilize theta/beta ratio in ADHD patients (Angelidis et al., 2016). Theta/beta ratio can predict ADHD with 94% sensitivity in childhood (Snyder and Hall, 2006). While neurotypical populations consistently present with a decrease in theta/alpha ratio with maturation.

Despite all the difficulties in making a diagnosis using EEG, devices using this method are being introduced into medical practice. Neuropsychiatric Electroencephalograph-Based ADHD Assessment Aid (NEBA) system received Food and Drug Administration approval (Food and Drug Administration, 2013).

In addition to the frequency range, when working with ADHD, it is reasonable to take into account the morpho-functional areas of the brain, which are responsible for executive functions, planning, decision-making, reward processing, and impulse control, such as prefrontal cortex, anterior cingulate cortex, and basal ganglia (Rubia et al., 2014).

Taken together, age is a critical factor influencing EEG signatures in ADHD. Theta-band abnormalities are more pronounced in children, while adults with ADHD often show alpha and beta dysregulation (Barry et al., 2010; Markovska-Simoska and Pop-Jordanova, 2017). These developmental shifts suggest that neurofeedback protocols should be age-tailored—targeting TBR (theta/beta ratio) in children and alpha/beta regulation in adults—to optimize therapeutic outcomes. Failure to adjust protocols may lead to reduced efficacy or delayed learning curves.

#### 3.3.3 Neurofeedback protocols with ADHD

Despite the fact that many studies have been devoted to the detection of morpho-functional symptoms of ADHD, these symptoms are unstable and heterogeneous across different age groups. As a consequence, the results of a particular therapy are verified solely by clinical symptoms.

There is currently no complete understanding of the basic principles of neurofeedback therapy. Moreover, there are a sufficient number of neurofeedback therapy protocols that are not standardized. However, some countries, such as Germany, the Netherlands, Austria and Russia, have implemented feedback techniques in patient care and provide partial or full budgetary financial support for feedback treatment of certain conditions. Overall, we can see a positive shift in some countries towards the acceptance of neurofeedback therapy for ADHD.

Currently, it is recommended that at least three double-blind placebo-controlled randomized controlled trials (RCTs) be conducted to verify the effectiveness of neurofeedback-based treatment of ADHD (Kuznetsova et al., 2023). However, the disadvantages of the ongoing studies are the small and heterogeneous sample of subjects and poorly standardized methods.

Lack of understanding of the mechanism of action of neurofeedback leads to underestimation of methods, since to assess effectiveness we need to know what specific parameters we should look at, taking into account individual and group variability and time-delayed dynamics of symptom reduction. Several articles argue that the successful outcome of feedback therapy depends on good self-regulation skills (Doehnert et al., 2008; Wan et al., 2014; Gevensleben et al., 2014; Strehl et al., 2017; Veilahti et al., 2021; Kuznetsova et al., 2023).

The most well-known neurofeedback treatment protocols are: neurofeedback implementing slow cortical potential (SCP), Z-Score neurofeedback protocol, theta/beta ratio (TBR) neurofeedback protocol, alpha/theta ratio neurofeedback protocol (A/T protocol), alpha/beta ratio neurofeedback protocol (A/B protocol), sensorimotor (SMR) neurofeedback protocol, and alpha neurofeedback protocol. Among the presented protocols, TBR and SMR are the most effective and frequently used for ADHD (Escolano et al., 2014; Marzbani et al., 2016; Arns et al., 2020; Enriquez-Geppert et al., 2019; Kuznetsova et al., 2023).

A/T protocol is focusing on raising the theta–alpha ratio in the EEG while the participant relaxes with eyes closed and auditory feedback of sounds aimed at elevating theta (4–7 Hz) over alpha (8–11 Hz). The origins of the A/T EEG neurofeedback protocol can be found in the pioneering efforts of Greene and Greene to gain control over the hypnagogic process and borderline conscious state conducive to creativity (Green and Green, 1977).

Besides increasing creativity the A/T protocol has shown its robust effectiveness in the treatment of conditions such as post traumatic stress syndrome (PTSD), alcoholism, depression, ADHD, anxiety disorders (Beck et al., 1961; Peniston and Kulkosky, 1991; Saxby and Peniston, 1995; Peniston and Kulkovsky, 1999; Spielberger et al., 1983). During this A/T protocol brain functioning involves the ascending mesencephalic-cortical arousal system, hippocampus and parahippocampal regions, and limbic circuits (O’Keefe and Nadel, 1978). This protocol seems simple enough, but it requires training the brain to switch between different frequency functional modes, which at first glance seems like a non-trivial task. It looks like the success of treating such conditions as ADHD, anxiety, obsession, depression, loss of concentration is associated with learning to break the neurophysiological dominant and to move into a hypnotic state. In fact, this protocol is learning self-hypnosis through feedback.

A/B protocol is focusing on relative spectral power (RSP) in alpha (8–11 Hz) and beta (18–30 Hz) bands in the EEG while the participant relaxes and concentrates with auditory feedback of sounds (Nicholson et al., 2023; Jurewicz et al., 2018).

The Z-Score neurofeedback protocol refers to a statistical measure that indicates how many standard deviations an individual’s brain activity deviates from the norm. Unlike traditional neurofeedback, which typically focuses on training specific frequency bands (e.g., theta, beta), Z-Score neurofeedback simultaneously assesses and trains multiple metrics of brain function and communication between different brain areas and compares an individual’s brain activity to a normative database of healthy brain function (Roy et al., 2020; Grosselin et al., 2021).

All the protocols are based on learning to initiate certain patterns of brain activity. All feedback exercises consist of 10–30 training sessions of 30 min each. Exercises are most often presented in a game form using a computer. The average course costs from $2,000 to $10,000 (Riesco-Matías et al., 2021; Van Doren et al., 2019; Bakhshayesh et al., 2011; Geladé et al., 2018).

### 3.4 Combined application of tDCS and neurofeedback

Emerging research supports the potential for combining transcranial direct current stimulation (tDCS) with neurofeedback to optimize treatment outcomes in ADHD. While tDCS facilitates neuroplasticity by modulating cortical excitability, neurofeedback trains individuals to self-regulate neural activity. This complementary mechanism suggests a synergistic interaction whereby tDCS may prime the brain to better respond to feedback-based learning. The rationale for combining neurofeedback with tES (including tDCS and tACS) lies in the modulation of both bottom-up and top-down mechanisms. Neurofeedback facilitates self-regulation by reinforcing desirable EEG patterns, whereas tES can directly influence the excitability and synchronization of cortical circuits. Integrating both approaches may create a feedback-enhanced neuromodulation loop, maximizing efficacy through complementary mechanisms.

Studies in other neuropsychiatric populations suggest potential benefits. Belkacem et al. (2023) demonstrated that combining real-time EEG feedback with tDCS modulates cortical plasticity more effectively than either approach alone. In ADHD, this could translate to greater gains in executive functions, particularly in populations resistant to monotherapies.

Preliminary work in other cognitive domains, such as learning and memory, supports this interaction. Reis et al. (2009) demonstrated enhanced motor learning following concurrent application of anodal tDCS and behavioral training. Similarly, Cohen Kadosh et al. (2012) showed that tDCS targeting the dorsolateral prefrontal cortex (DLPFC) potentiated the effects of cognitive training. These findings imply that tDCS may increase cortical responsiveness to neurofeedback cues, accelerating skill acquisition and possibly enhancing long-term retention.

To date, limited but promising studies have explored this combination in ADHD. Belkacem et al. (2023) proposed a closed-loop system integrating EEG-based feedback with brain stimulation to optimize real-time neuromodulation. Future studies should evaluate whether sequential or concurrent administration of tDCS and neurofeedback leads to superior improvements in attentional control, executive function, and symptomatology in ADHD. We recommend future research also to explore the timing, dosage, and personalization of these combined interventions, ideally guided by individual neurophysiological profiles and computational models.

Beyond hardware interventions like tDCS and neurofeedback, a robust body of evidence supports cognitive training and behavioral interventions. Cortese et al. (2015) conducted a meta-analysis on cognitive training, showing moderate effects on working memory (Hedges’ g = 0.52) but limited generalization to core ADHD symptoms. However, combination with other modalities may yield greater benefits.

Arns et al. (2020) reviewed neurofeedback interventions and rated them as “Level 1 – Efficacious and Specific” for ADHD, particularly SMR and TBR protocols. Their findings emphasize the importance of treatment personalization and adherence to standard protocols across sessions to optimize outcomes.

Additionally, behavioral therapies such as cognitive training, executive function coaching, and computerized attention programs (e.g., Cogmed, ACTIVATE) show domain-specific benefits. These are especially useful in younger populations and may augment or maintain gains achieved through neuromodulation.

## 4 Conclusion

To sum up, tDCS seems to be a reliable method for improving ADHD deficits both in adults and children. While performance of HD-tDCS is comparable to conventional tDCS, future studies need to investigate if further personalization of the stimulation protocols (based on individual MRI and electric field calculations) can significantly improve the effect. Additionally, it would be beneficial for future studies to explore the long-term effects of tDCS on ADHD symptoms and whether it can be used as a standalone treatment or in combination with other interventions (see Table 2). Overall, tDCS shows potential as a non-invasive and safe method for addressing ADHD deficits, but further research is needed to optimize its effectiveness.

**TABLE 2:** Transcranial direct current stimulation (tDCS) and electroencephalography (EEG)-based brain-computer interfaces (BCI) comparison.

	Pharmacological intervention	HD-tDCS	tDCS	EEG-BCI
Safety	Relatively safe	Relatively safe	Relatively safe	Safe
Efficiency	Distinct effects	No effects, distinct effects	Distinct effects	No effects, distinct effects
Long-term effect	Primary adhd symptoms: enhancing concentration and diminishing impulsiveness	Reduced commission errors after 6 weeks inverventions (Wang et al., 2023); Reduced omission errors after 5 days stimulation (Breitling-Ziegler et al., 2021)	Partial improving effects on cognitive deficits (response inhibition, working memory, attention, and cognitive flexibility) and clinical symptoms (e.g., impulsivity and inattention)	Working memory, attention, cognitive enhancement
Comfort/side effects	Methylphenidate increased the risk ratio (rr) of serious adverse events (rr 1.36); any psychotic disorder (rr 1.36); and arrhythmia (rr 1.61) compared to no intervention (Storebø et al., 2015); Potential risk of abuse; long-term administration can lead to growth retardation (decreased height, weight, and bone marrow density)	Burning or tingling sensations; metallic taste in the mouth; headaches	Burning or tingling sensations; metallic taste in the mouth; headaches	Comfort
Age group	6–20	8–18	8–18	> 6
Contraindications	Methylphenidate: monoamine oxidase inhibitors (maois) intake;glaucoma, severe hypertension, motor tics, tourette syndrome, or a family history of tourette syndrome	Metal device implants (such as the cochlear implant, the artery clamp, and the pacemaker); history of brain trauma or cerebrovascular accident, intracranial hypertension, skull defects, epilepsy, and other serious neurological, circulatory, endocrine, and other physical diseases; audio-visual impairments and color blindness, color weakness, or narrow-angle glaucoma	Metal device implants (such as the cochlear implant, the artery clamp, and the pacemaker); history of brain trauma or cerebrovascular accident, intracranial hypertension, skull defects, epilepsy, and other serious neurological, circulatory, endocrine, and other physical diseases; audio-visual impairments and color blindness, color weakness, or narrow-angle glaucoma	–
Perspective	Growing number of new pharma remedies	Different current intensities and target areas	Different target areas	Directed neuroplasticity

Considering the research on the use of neurofeedback for the ADHD treatment, we can conclude that the method is verified in terms of safety and simplicity, but requires more detailed study and standardization. TDCS’s non-invasive approach combined with neurofeedback’s long-term plasticity potentially could result in a more distinct effect for reducing ADHD symptoms.

## Footnotes

1
https://icd.who.int/
